# Molecular mechanisms in podocytopathies: finding suitable targets for a new era of glomerular gene therapy

**DOI:** 10.1093/ckj/sfaf332

**Published:** 2025-11-03

**Authors:** Nadia Tavakolidakhrabadi, Wen Y Ding, Gavin I Welsh, Moin A Saleem

**Affiliations:** Bristol Medical School, Bristol Renal, University of Bristol, Dorothy Hodgkin Building, Whitson Street, Bristol, UK; Bristol Medical School, Bristol Renal, University of Bristol, Dorothy Hodgkin Building, Whitson Street, Bristol, UK; Bristol Medical School, Bristol Renal, University of Bristol, Dorothy Hodgkin Building, Whitson Street, Bristol, UK; Bristol Medical School, Bristol Renal, University of Bristol, Dorothy Hodgkin Building, Whitson Street, Bristol, UK; Bristol Royal Hospital for Children, Department of Paediatric Nephrology, Bristol Royal Hospital for Children, Upper Maudlin Street, Bristol, UK

**Keywords:** AAV, CRISPR, gene therapy, glomerular filtration barrier, podocytopathies

## Abstract

Podocytopathies encompass a spectrum of glomerular disorders driven by structural and functional impairments in podocytes: specialized cells critical for maintaining the glomerular filtration barrier. Dysregulation of key podocyte components—such as slit-diaphragm proteins (nephrin, podocin), cytoskeletal regulators (ACTN4, TRPC6), and adhesion complexes (integrins, dystroglycan)—leads to proteinuria and progressive glomerulosclerosis. Current therapies often fail to address underlying genetic or molecular defects, particularly in hereditary or refractory cases. Gene therapy has emerged as a transformative approach, leveraging adeno-associated viral (AAV) vectors, CRISPR-based editing, and RNA modulation to correct pathogenic mutations or restore disrupted pathways. Recent advances in capsid engineering, tissue-specific promoters, and delivery strategies have enhanced podocyte targeting while minimizing off-target effects. Preclinical successes, including AAV-mediated rescue of NPHS2-associated nephrotic syndrome and complement modulation in IgA nephropathy, highlight the therapeutic potential. However, challenges such as immune responses, vector biodistribution, and disease heterogeneity remain. This review synthesizes the molecular mechanisms underlying podocytopathies, evaluates current gene-therapy strategies, and discusses translational hurdles and future directions, including patient-derived organoid models and combinatorial therapies. By bridging mechanistic insights with innovative gene-based interventions, this work underscores the promise of precision medicine in revolutionizing the treatment of podocytopathies.

## INTRODUCTION

**Figure 1:**
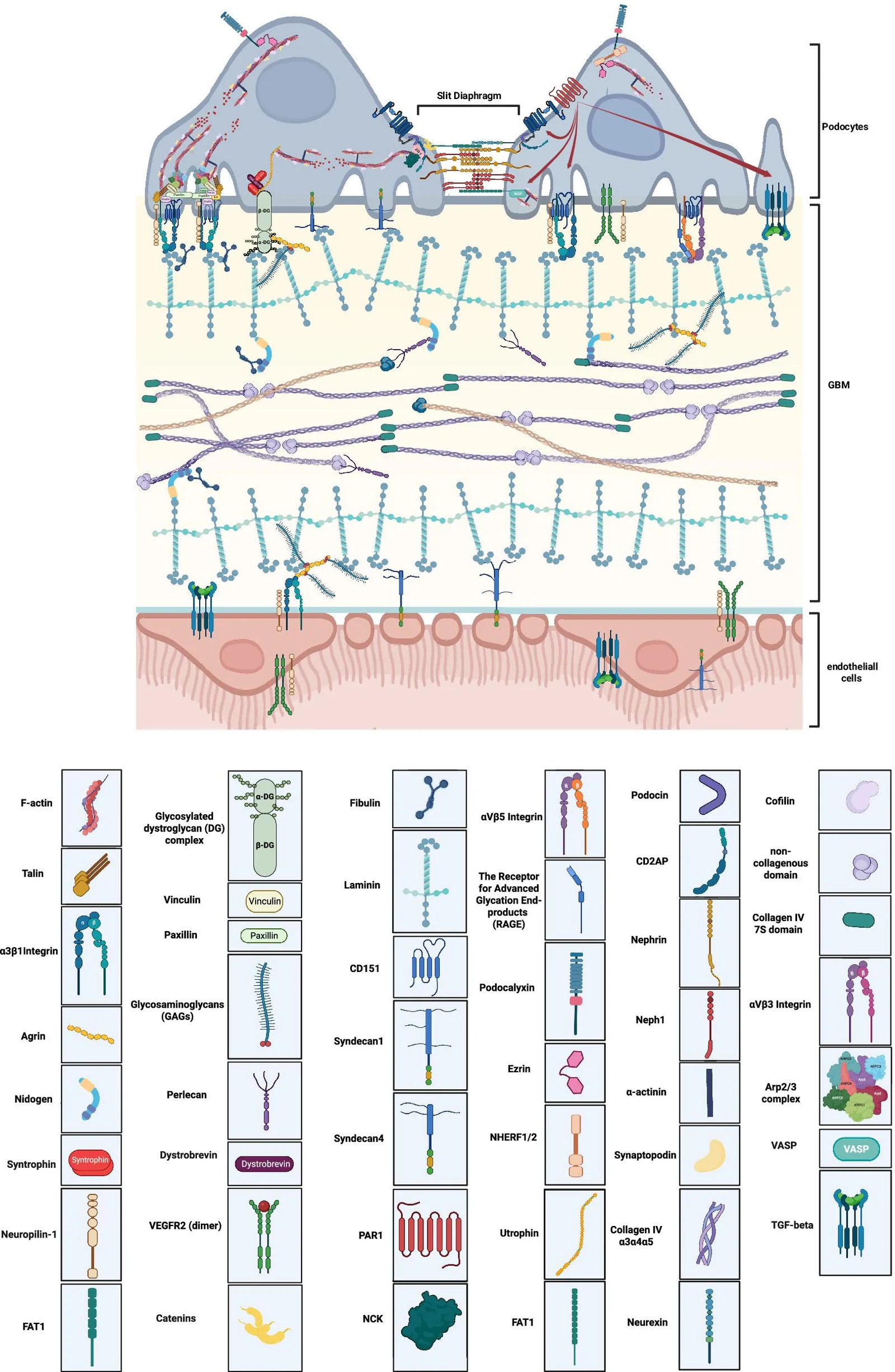
Molecular landscape of the GBM and associated podocyte interface: adhesion, cytoskeletal regulation, and extracellular matrix architecture. This figure illustrates the intricate molecular network at the podocyte–GBM interface, highlighting key structural, signalling, and cytoskeletal components critical for maintaining the GFB.

Podocytopathies represent a heterogeneous group of glomerular disorders characterized by structural or functional impairment of podocytes, highly specialized epithelial cells critical for maintaining the glomerular filtration barrier (GFB) [1]. These cells exhibit a complex architecture, with interdigitating foot processes anchored by specialized slit-diaphragm proteins such as nephrin (encoded by *NPHS1*) and podocin (encoded by *NPHS2*) (Fig. 1) [2]. Podocyte injury disrupts the GFB, leading to proteinuria, progressive glomerulosclerosis, and eventual chronic kidney disease (CKD) [3]. Current therapeutic strategies, including immunosuppression [4] and renin–angiotensin system inhibition [5], often fail to address the underlying genetic or molecular defects underlying disease, particularly in hereditary or refractory cases [6]. Gene therapy has emerged as a transformative approach to target the root causes of podocyte dysfunction, offering potential cures through gene replacement, genome editing, or RNA-based modulation [6–8]. This review synthesizes current knowledge on the molecular underpinnings of podocytopathies, in preparation for advances in precision medicine and the relevant translational challenges.

## KEY DETERMINANTS OF PODOCYTES HEALTH

The GFB (300–350 nm) is a trilaminar capillary wall comprising fenestrated endothelium, a fused glomerular basement membrane (GBM), and interdigitating podocyte foot processes [9].

Podocyte architecture relies on a specialized cytoskeleton tightly regulated by actin-associated proteins; primary processes are rich in microtubules, while foot processes contain bundled and cortical F-actin networks [10]. Each foot process is anchored by two structural hubs; focal adhesions (FAs) at the basal membrane and slit diaphragms at the cell–cell contacts, which link the actin core to external structures [10]. F-actin, the fundamental structural filament of podocyte foot processes, underpins their shape and motility. Synaptopodin promotes the formation and stabilization of actin stress fibres and prevents degradation by suppressing cathepsin L activity. Conversely, cofilin enhances actin turnover through filament severing and depolymerization, enabling the podocyte to respond dynamically to stress and injury [11].

### The podocyte slit diaphragm

Between adjacent podocyte feet, a specialized slit diaphragm is built from nephrin, Neph1 (Neph family), and podocin, together with tight junction components (e.g. P-cadherin, ZO-1, FAT1) [10]. The slit diaphragm behaves as a dynamic hub: its architecture sets slit size, and its regulation of podocyte signalling continuously adjust actin tension and foot-process morphology to maintain selective filtration. The slit-diaphragm complexes link to the actin cytoskeleton and forms a signalling scaffold that maintains the slit architecture. Mutations and/or dysregulation in nephrin, podocin or related components cause foot-process effacement and proteinuria [12]. Nephrin, a transmembrane protein of the immunoglobulin superfamily, and Neph1 extends into the filtration slit and interacts homophilically and heterophilically with neighbouring nephrin molecules to form a size- and charge-selective barrier. Intracellularly, nephrin recruits the cytoskeletal adaptor proteins CD2AP and Nck (Non-catalytic region of tyrosine kinase), which anchors the complex to the actin cytoskeleton and facilitates downstream signalling. Podocin, a membrane-associated protein, stabilizes nephrin within lipid rafts and modulates the activity of TRPC6 calcium channels, thereby regulating actin dynamics and cytoskeletal organization. The slit diaphragm also functions as a signalling hub, integrating mechanical and chemical cues to maintain the integrity of the filtration barrier. For example, nephrin phosphorylation promotes actin polymerization, while CD2AP interacts with PI3K—a phosphoinositide 3-kinase involved in intracellular signalling—and other cytoskeletal regulators to support podocyte structure and function [10, 12, 13].

Importantly, podocyte VEGF signalling feeds into the slit diaphragm: autocrine VEGF‐A (via VEGFR2/neuropilin-1 coreceptors secreted by podocytes) upregulates podocin and enhances podocin–CD2AP interaction, reinforcing slit-diaphragm integrity [14].

### The podocyte–basement membrane interface

At the podocyte–GBM interface, laminin-binding integrin receptors (especially α3β1) and the dystroglycan complex on foot processes bind the laminin-rich GBM, anchoring podocytes to the membrane and transmitting mechanical cues [9, 15]. Integrin receptors (especially laminin-binding α3β1) cluster at FAs and recruit adaptor proteins such as talin, vinculin, paxillin, and other focal adhesion-associated molecules including the ILK–PINCH–Parvin complex. to couple the extracellular matrix (ECM) to F-actin [10, 13]. These adhesion complexes not only mechanically stabilize foot processes but also organize signalling nodes (e.g. FAK, Src, p130Cas) that regulate actin dynamics. In health, tension from myosin IIA in the cell body generates strain that strengthens integrin–actin linkages and maintains slit spacing [10, 13]. In disease, disruption of talin/vinculin/paxillin linkages or calpain-mediated cleavage leads to actin disorganization, foot‐process effacement, and proteinuria [10, 13]. Likewise, the fenestrated endothelial cells adhere to the GBM via laminin-binding integrins (and possibly dystroglycan), linking the endothelial glycocalyx to the underlying basement membrane [15]. The tetraspanin CD151 associates tightly with α3β1, strengthening the podocyte–matrix link: human CD151 mutations cause proteinuria, and *Cd151⁻/⁻* mice develop stress-induced glomerular injury [16]. Other integrins (including α1β1, α2β1) may engage collagen or fibronectin matrices. In parallel, transmembrane proteoglycans (syndecan-1/4) and the dystroglycan complex provide auxiliary attachments [13]. Syndecans cooperate with integrins to form adhesion foci and modulate receptor trafficking; for example, podocyte-specific loss of heparan-sulfate chains (EXT1 knockout) alters adhesion size and F-actin organization, often upregulating surface syndecan-4 without causing overt proteinuria [13]. Dystroglycan’s extracellular α-subunit binds GBM components such as laminin and agrin, while the intracellular β-subunit connects to utrophin (a dystrophin homolog), which in turn associates with dystrobrevin and syntrophin to link the complex to F-actin and signalling enzymes [13, 17]. Immunolocalization studies reveal the presence of utrophin and dystroglycan at the podocyte basal surface, where they co-localize with laminin and agrin in the GBM. This spatial arrangement suggests that, as in muscle tissue, these proteins form a dystrophin–glycoprotein complex that links the intracellular actin cytoskeleton to the extracellular matrix, indicating a conserved structural role across cell types [17]. Thus, a multilayered adhesion network (integrins/CD151–talin/vinculin/paxillin; syndecans; dystroglycan–utrophin/dystrobrevin/syntrophin) anchors the podocyte cortex to the GBM scaffold and transduces biomechanical signals.

The GBM itself is a dense extracellular matrix network of type IV collagen (predominantly the α3α4α5 heterotrimer), which contains characteristic non-collagenous (NC1) and 7S domains serving as binding sites for integrins and the DG complex and facilitating bidirectional communication between podocytes and the ECM, and laminin (principally LM-521), with nidogen crosslinks and abundant heparan-sulfate proteoglycans (HSPGs), chiefly agrin and perlecan, secreted by podocytes and endothelium [13, 15, 18]. This composite matrix provides a highly selective sieve: the interwoven collagen/laminin layers impose size-selectivity, while the negatively charged glycosaminoglycan chains (from agrin and other HSPGs) create an electrostatic barrier that repels anionic macromolecules and binds to both growth factors and matrix proteins, helping organize the GBM architecture [9, 12, 13, 15–17].

Apically, the sialomucin podocalyxin (PC) forms another key complex. PC carries a heavy negative charge that helps separate foot processes, and its cytoplasmic tail contains a PDZ-binding motif that recruits NHERF1/2. NHERF proteins then bind ERM proteins (ezrin/moesin), creating a tripartite PC–NHERF–ezrin complex that anchors PC to F-actin [19]. This linkage maintains the cuboidal podocyte shape and open filtration slits. Disruption of this complex (by phosphatase activation or charge neutralization) causes PC to uncouple from actin, coinciding with foot-process fusion [19]. At focal adhesions, scaffolds such as talin, vinculin, and paxillin similarly serve as modular platforms: paxillin’s LD and LIM domains bind vinculin, FAK, Src, and other adhesion-associated kinase, coordinating actin anchoring with kinase activity [13].

This coordinated assembly of slit-diaphragm junctions, cell–matrix adhesion complexes, and the specialized GBM matrix is essential for the glomerular structural integrity and perm-selectivity [9, 12]. The GBM is actively remodelled via podocyte-derived matrix metalloproteinases (MMPs) and regulated by tissue inhibitors of metalloproteinases (TIMPs). Structural and regulatory components, including endostatin, contribute to ECM integrity and signalling. This highly organized matrix supports filtration, sustains biomechanical forces, and governs podocyte behaviour essential for glomerular function [20].

### Podocyte signalling receptors

Podocyte function is also shaped by signalling receptors that sense extracellular cues. Notably, podocytes express VEGF receptors: VEGFR2 (flk-1) is upregulated on differentiation, and neuropilin-1 (NP1) serves as a VEGF/semaphorin coreceptor [14]. Podocyte-derived VEGF in turn acts in an autocrine/paracrine loop: VEGFR2 activation promotes cell survival, and as noted above drives podocin/CD2AP assembly at the slit diaphragm [14]. Beyond growth factors, podocytes are sensitive to protease signals: protease-activated receptor 1 (PAR1) on podocytes responds to thrombin and other serine proteases, which are thought to be upregulated in the disease states [21]. In diabetic and focal segmental glomerulosclerosis (FSGS) models, excessive PAR1 stimulation causes podocyte Ca^2+^ overload through TRPC6 channels, which induces cytoskeletal contraction and therefore foot-process retraction, and apoptosis [22]. Previously, it has been shown that PAR1 overactivation lead to heightened phosphorylation of vasodilator-stimulated protein, hence affecting podocytes’ actin cytoskeleton and motility [21]. Activated TGF-β signalling not only drives podocyte apoptosis and dedifferentiation through inducing Smad7 and p38 MAPK–dependent caspase-3 activation, causing podocyte death [23], but also promotes stress-fibre formation and epithelial–mesenchymal transition of podocytes while upregulating extracellular matrix proteins (collagens, CTGF) [24]. Elevated TGF-β can also potentially suppress VEGF expression, compounding endothelial and podocyte dysfunction [25], while PAR1 activation can amplify injury by exacerbating calcium overload and cytoskeletal collapse. Together, their interplay creates a feed-forward loop of podocyte damage, foot-process effacement, and proteinuria characteristic of glomerular diseases such as FSGS and diabetic nephropathy. In addition, the receptor for advanced glycation end products (RAGE) is expressed at low levels basally but becomes upregulated in hyperglycaemia [26]. AGE–RAGE binding stimulates NF-κB and oxidative signalling in podocytes, elevating VEGF and inflammatory mediators. In mouse diabetes models, RAGE blockade (or gene deletion) markedly reduces VEGF-driven inflammation, GBM thickening, and albuminuria [20, 26].

### Signalling pathways downstream of circulating factors

Structural integrity and cytoskeletal dynamics are paramount for podocyte function, and several molecules converge to regulate these processes. ACTN4 (α-actinin-4) [27], an actin-binding protein, and PDLIM1 (PDZ and LIM domain protein 1), which links actin filaments to signalling pathways [28] are critical for maintaining the organization of the actin cytoskeleton, and even minor disturbances in their expression or function can compromise the stability of the foot processes. In the context of minimal change disease (MCD), where the architecture is disrupted yet not irreversibly lost, such perturbations might contribute to the transient effacement observed under electron microscopy. Furthermore, the transcriptional landscape that governs podocyte phenotype is finely tuned by regulators such as CDKN1A (p21) and LMX1B. While CDKN1A can enforce cell cycle arrest in these terminally differentiated cells [29], LMX1B orchestrates the expression of essential slit-diaphragm components, ensuring that subtle alterations in transcriptional control may predispose podocytes to injury without causing cell death [30, 31].

Signalling pathways play a pivotal role in mediating podocyte responses to injury. The mechanistic target of rapamycin (MTOR) pathway, for instance, is central to regulating cell growth, metabolism, and autophagy, and dysregulated mTOR signalling can lead to cytoskeletal abnormalities that favour foot-process effacement [32, 33]. Similarly, the p38 MAPK family acts as a stress-responsive kinase system, translating inflammatory and oxidative signals into cellular responses that further destabilize the actin cytoskeleton [34, 35]. In parallel, calcineurin signalling, mediated by its catalytic subunits (PPP3CA, PPP3CB, and PPP3CC) [36] and modulated by FKBP1A [37], is critical in controlling the activity of nuclear factor of activated T cells (NFAT) [38]. This pathway is especially relevant given the well-established efficacy of calcineurin inhibitors in MCD, which are thought to stabilize podocyte structure by dampening aberrant dephosphorylation events and preserving actin organization [39, 40].

The regulation of nucleotide synthesis and cell proliferation is also implicated in podocyte stability. IMPDH1 and IMPDH2, which are key enzymes in the de novo synthesis of guanine nucleotides, have become targets for immunosuppressive therapies such as mycophenolate mofetil [41–43]. By limiting T-cell proliferation, the inhibition of IMPDH might indirectly protects podocytes from the cytokine milieu that drives the characteristic reversible injury of MCD [44, 45]. Furthermore, the glucocorticoid receptor (NR3C1) is central to the therapeutic response in MCD; glucocorticoids exert their beneficial effects by binding to NR3C1, thereby modulating gene expression and attenuating inflammatory responses that contribute to podocyte damage [46, 47].

Extracellular mediators and modulators further refine the podocyte response in MCD. Clusterin (CLU) acts as a chaperone protein with roles in lipid transport and apoptosis regulation, and its upregulation may represent a protective response to cellular stress [48, 49]. Similarly, CMIP (c-Maf inducing protein) has been shown to be upregulated in MCD, where it disrupts the stability of the nephrin–podocin–CD2AP complex, leading to podocyte cytoskeletal disorganization and foot-process effacement [50]. The cytokine transforming growth factor beta 1 (TGFB1) is another important player; although it is more often associated with fibrotic processes, low-level TGF-β1 signalling may contribute to subtle changes in podocyte structure without triggering overt sclerosis [51, 52]. Moreover, the vitamin D receptor (VDR) modulates both immune responses and cellular differentiation [53, 54]. Enhanced VDR signalling can protect podocytes by inhibiting detrimental renin–angiotensin system activation and reducing pro-inflammatory cytokine production [55–57]. Finally, surfactant proteins such as SFTPA1 and SFTPD, while traditionally associated with pulmonary function [58], possess immunomodulatory properties that might influence local inflammatory responses within the glomerulus, thereby indirectly affecting podocyte stability [58].

MCD is characterized by the reversible effacement of podocyte foot processes, a process driven by complex alterations in many molecular pathways [59–61]. At the metabolic and immunomodulatory level, enzymes involved in γ-aminobutyric acid (GABA) metabolism—such as ABAT (GABA transaminase), ALDH5A1 (succinate semialdehyde dehydrogenase), and GAD2 (glutamate decarboxylase)—are traditionally associated with neurotransmitters regulation but are also expressed in non-neuronal tissues, including the kidney [62]. These enzymes can modulate immune cell activity by influencing T-cell responses, macrophage activation, and cytokine secretion [63], thereby shaping the local microenvironment in which podocytes reside. This immune modulation may contribute to podocyte injury by altering cytoskeletal dynamics, disrupting foot-process architecture, and activating inflammatory signalling pathways [64]. Concomitantly, the proper function of detoxification systems—exemplified by GSTM1 (glutathione *S*-transferase mu 1) and GSTT1 (glutathione *S*-transferase theta 1)—ensures efficient neutralization of reactive oxygen species and other metabolic by-products, thereby protecting podocytes from oxidative damage that could exacerbate foot-process effacement [65].

All these molecules delineate a complex network of metabolic, cytoskeletal, transcriptional, and signalling pathways that converge to determine podocyte function and the development of MCD. The transient nature of foot-process effacement in MCD reflects a finely balanced interplay between injury and repair mechanisms, with a predominant contribution from immune dysregulation and its downstream effects on the actin cytoskeleton. Understanding the precise roles of these molecules not only enhances our comprehension of MCD pathogenesis but also paves the way for targeted therapeutic interventions that could stabilize podocyte structure and restore glomerular filtration integrity.

## CLASSIFICATION AND PATHOGENESIS OF PODOCYTOPATHIES

### Primary podocytopathies

#### Minimal change disease 

MCD is the most common cause of nephrotic syndrome in children—a clinical condition characterized by heavy proteinuria, hypoalbuminemia, oedema, and hyperlipidaemia—and is histologically defined by diffuse podocyte foot-process effacement on electron microscopy without significant glomerular inflammation or scarring [66]. MCD is usually termed idiopathic, without a known underlying cause, with occasional familial cases described [67, 68]. While traditionally considered an immune-mediated disorder linked to T-cell dysregulation and cytokine release (e.g. interleukin-13 or angiopoietin-like-4) [69], recent evidence suggests that pathogenesis could be B-cell driven. For example, Rituximab, an anti-CD20 monoclonal antibody targeting B cells, has been a highly effective treatment for MCD [70, 71]. Although there is some evidence that Rituximab has direct effects on the podocyte via preservation of sphingomyelin phosphodiesterase acid-like 3b (SMPDL-3b) [72], there is increasing evidence for B-cell involvement in disease. Recently anti-nephrin antibodies have been shown to correlate with proteinuria in MCD, with Rituximab administration correlating with decreased proteinuria and decreased anti-nephrin antibodies [73]. In rodent models, administration of the monoclonal antibody 5-1-6, which targets the extracellular domain of nephrin, induces massive yet reversible podocyte FP effacement and proteinuria, effectively recapitulating MCD phenotype. A single IV dose leads to peak proteinuria by days 5–8 with full resolution by approximately day 18, all in the absence of complement activation or inflammatory cell infiltration—a hallmark of MCD-like injury [74]. This precise targeting of slit-diaphragm nephrin demonstrates that disruption of nephrin alone is sufficient to cause acute, reversible podocyte dysfunction, establishing a robust and specific animal model for studying antibody-mediated glomerular disease. In addition, anti-slit-diaphragm antibodies on kidney biopsy have been identified in the absence of anti-nephrin antibodies [75], which might suggest that more than one circulating antibody could be responsible for MCD. Despite high glucocorticoid responsiveness in both adults and children (90%) [76], MCD often follows a relapsing course [77], underscoring the need for therapies targeting podocyte-specific pathways (Table 1).

**Table 1:** Histological features of nephrotic syndrome subgroups. This table summarizes representative clinical entities, genetic mechanisms, histological findings, and newly added clinical context, including epidemiology, typical disease course, and therapeutic implications. Monogenic forms are typically early-onset and steroid-resistant, representing the most viable candidates for gene-therapy approaches.

Subgroup	Representative entities	Genetics and pathogenesis	Histologic pattern	Epidemiology, clinical course, and therapeutic implications
Idiopathic	MCD, primary FSGS, collapsing glomerulopathy	Circulating permeability factors (e.g. anti-nephrin antibodies), T-cell-mediated injury	Diffuse foot-process effacement; FSGS variants	Most common in children and adults; MCD often steroid-sensitive with good prognosis, whereas primary FSGS may show variable steroid responsiveness and relapse; immunosuppression remains standard therapy.
Monogenic (Mendelian)	Denys–Drash syndrome: *WT1* mutations	Heterozygous *WT1* missense/nonsense variants → defective podocyte differentiation and nephrin expression [78]	Diffuse mesangial sclerosis (DMS)	Typically presents in infancy or early childhood; steroid-resistant; progresses rapidly to ESKD; gene therapy represents the most rational curative strategy.
	Pierson syndrome: *LAMB2* mutations	Biallelic *LAMB2* loss-of-function → abnormal laminin β2 in GBM → impaired podocyte–GBM adhesion [79]	Congenital NS with mesangial expansion (DMS-like)	Congenital presentation; severe, treatment-resistant; limited response to immunosuppression; potential candidate for targeted gene replacement.
Congenital/infantile NS	*PLCE1* mutations	Recessive or dominant *PLCE1* mutations disrupting podocyte signalling pathways	DMS or FSGS depending on mutation class	Early childhood onset; some variants may show partial steroid response; overall poor prognosis; targeted gene therapy may restore signalling balance.
	*NPHS1* mutations	Biallelic *NPHS1* loss-of-function → absent nephrin → congenital NS with podocyte dysregulation	Mesangial proliferation, immature glomeruli with interstitial involvement and foot-process effacement	Commonest cause of congenital NS in Finland and other founder populations; steroid-resistant; treated with supportive care; excellent model for AAV- or mRNA-based gene replacement.
	Other genes (including *NPHS2*, *ACTN4*, *TRPC6*, *INF2*, *COQ2–8*, *CRB2*)	Mutations affecting slit-diaphragm, cytoskeletal, mitochondrial, or adhesion proteins; collectively account for ≈two-thirds of congenital and infantile NS cases	Predominantly FSGS or FSGS-like lesions	Variable onset (infantile to adult); typically, steroid-resistant; supportive care or transplantation required; gene-based or protein replacement therapies hold promise.

NS, nephrotic syndrome.

**Table 2:** Summary of major monogenic podocytopathies and their corresponding OMIM identifiers. The table summarizes representative genes responsible for congenital, infantile, or familial steroid-resistant nephrotic syndrome, indicating their encoded proteins, inheritance modes, and major associated disease entities. These genes collectively contribute to the spectrum of hereditary podocyte disorders leading to FSGS or diffuse mesangial sclerosis.

Gene	Encoded protein/function	Inheritance	Associated disease/syndrome	OMIM ID(s)
*NPHS1*	Nephrin (slit-diaphragm protein)	AR	Finnish-type congenital nephrotic syndrome	602716/256300
*NPHS2*	Podocin (slit-diaphragm protein)	AR	FSGS, steroid-resistant nephrotic syndrome	604766/603278
*WT1*	Transcription factor (podocyte differentiation)	AD	Denys–Drash syndrome/Frasier syndrome	607102/194080/136680
*LAMB2*	Laminin β2 (GBM adhesion)	AR	Pierson syndrome	150325/609049
*PLCE1*	Phospholipase Cε1 (podocyte signalling)	AR/AD	Early-onset nephrotic syndrome, DMS/FSGS	608414/616026
*TRPC6*	Cation channel (Ca²⁺ homeostasis)	AD	Familial FSGS	603652/603965
*ACTN4*	α-Actinin-4 (cytoskeletal stability)	AD	Familial FSGS	604638/603278
*INF2*	Inverted formin-2 (actin polymerization)	AD	FSGS/Charcot–Marie–Tooth neuropathy E	610982/614082
*MYO1E*	Non-muscle myosin 1E	AR	FSGS	601479/614131
*LMX1B*	Transcription factor	AD	Nail–Patella syndrome	602575/161200
*CD2AP*	Scaffolding protein (nephrin–actin link)	AD	FSGS	604241/609046
*APOL1*	Apolipoprotein L1	Risk alleles	APOL1-associated FSGS	603743/612551

**Table 3:** Secondary podocytopathies. This table outlines key subgroups contributing to secondary FSGS, highlighting representative causes or genetic susceptibilities, underlying mechanisms of podocyte injury, and characteristic histopathologic features.

Subgroup	Examples and genetics	Mechanism	Histologic pattern	Epidemiology, clinical course, and therapeutic implications
Genetic susceptibility	APOL1 high-risk alleles (G1/G2)	Non-Mendelian risk variants → gain-of-function ion channels activated by interferon/infection	Secondary FSGS or collapsing glomerulopathy	Predominant in individuals of African ancestry; variable age of onset; often steroid-resistant and progressive; supportive care and RAAS blockade are mainstays; gene-silencing or editing strategies may provide future therapeutic benefit.
Metabolic/hemodynamic stress	Diabetes, obesity, hypertension	Hyperfiltration, oxidative/ER stress, podocyte hypertrophy	Perihilar FSGS, foot-process effacement	Highly prevalent in adults; gradual progression; managed through glycaemic, blood-pressure and lipid control; responds to SGLT2 inhibitors and RAAS blockade; gene therapy unlikely to play a major role.
Immune-mediated	SLE, ANCA vasculitis, HIVAN, membranous nephropathy	Immune-complex deposition, cytokine-mediated cytotoxicity	Immune-complex GN patterns; FSGS variants	Affects both children and adults; often relapsing–remitting; generally responsive to corticosteroids or other immunosuppressants; targeted biologics under investigation; gene therapy not currently indicated.
Drugs/toxins	Interferons, pamidronate, calcineurin inhibitors, heroin	Direct podocyte toxicity → apoptosis, cytoskeletal disruption	Collapsing FSGS or classic FSGS	Occurs secondary to exposure; may resolve after withdrawal of offending agent; supportive management; prevention via drug monitoring and avoidance of nephrotoxic agents.
Depositional disorders	AL amyloidosis, LCDD, cryoglobulinemia	GBM/mesangial distortion by protein deposits → podocyte detachment	Mixed nephrotic patterns; secondary FSGS scars	Typically adult-onset; progressive course; therapy directed at underlying plasma-cell or immune disorder (e.g. chemotherapy, immunomodulation); gene therapy not applicable.
Adaptive response	Hypoplasia, reflux nephropathy, nephrectomy, obesity	Compensatory hyperfiltration → podocyte enlargement and eventual detachment	Segmental sclerosis (perihilar)	Seen across age groups in reduced-nephron settings; slowly progressive; responds to RAAS blockade and blood-pressure control; emphasis on prevention of hyperfiltration injury.

#### Focal segmental glomerulosclerosis 

FSGS is a histological diagnosis that is characterized by the progressive scarring of the glomeruli, which impairs kidney function. It is a heterogeneous condition, often divided into primary and secondary forms. The cause of primary FSGS is uncertain, but one hypothesis is that underlying mechanisms might overlap with that of MCD. Monogenic forms are one distinct cause and have a high prevalence when it presents early (60%–70% in those presenting age under 3 months) but decreases with age [80]. Monogenic FSGS has been reported in 21.4% of patients with onset of disease between 19 and 25 years of age [81]. In the DUPLEX FSGS study, which recruited patients with a median age of 42 years (90.6%, >18 years of age), 33 patients out of the 352 available samples had a pathogenic genetic variant (9.4%) in genes integral to podocyte function and structure, while 27 patients (7.7%) had a pathogenic collagen 4 variant and another 13 patients (4%) had high-risk *APOL1* genotypes. Understanding the roles of these genetic and molecular factors is crucial for unravelling the disease’s pathogenesis and for identifying potential therapeutic targets.

Elevated levels of TGFB1, a potent pro-fibrotic cytokine, play a central role in the pathogenesis of FSGS [82]. TGFB1 induces mesangial cell activation, podocyte apoptosis, and deposition of extracellular matrix components, contributing to glomerulosclerosis and progressive kidney damage [83, 84].

The complement system, particularly C3AR1, is involved in the immune-mediated pathology of FSGS [85]. Activation of C3AR1 leads to inflammatory signalling that results in podocyte injury, apoptosis, and mesangial expansion, exacerbating glomerulosclerosis [86].

These findings reveal the complex molecular landscape of FSGS, where immune activation, inflammatory pathways or mutations in genes affecting podocyte structure, function, and signalling contribute to glomerular injury and sclerosis. Understanding these molecular mechanisms offers valuable insights into potential therapeutic strategies for preventing and treating FSGS.

#### Collapsing glomerulopathy 

Collapsing glomerulopathy (CG) is a severe variant of FSGS, marked by the collapse of glomerular capillary tufts and proliferation of overlying podocytes. It is associated with rapid progression to end-stage renal disease and is often resistant to conventional therapies [87].

Vascular Endothelial Growth Factor A (VEGFA) is pivotal in maintaining the GFB’s integrity, primarily through its expression in podocytes [88]. Studies have demonstrated that the overexpression of VEGFA can contribute to CG pathogenesis [89]. Specifically, podocyte-specific overexpression of the VEGFA164 isoform in mice induces a phenotype resembling CG, characterized by podocyte proliferation and capillary loop collapse [88]. Conversely, reduced VEGFA expression has been observed in collapsing glomeruli from patients with HIV and COVID-19, suggesting that downregulation may also play a role in disease development [90].​

ARMH4 (Armadillo Repeat Containing 4), also known as C14orf37 or UT2, is a podocyte-enriched protein implicated in CG [91]. Transcriptomic analyses have revealed significant downregulation of ARMH4 in glomerular diseases, including CG and classic FSGS. Immunohistochemical studies corroborate these findings, showing reduced ARMH4 protein levels in diseased glomeruli compared with normal tissue. Functionally, ARMH4 modulates inflammatory responses in podocytes; its overexpression suppresses the release of pro-inflammatory cytokines such as IL-1β and IL-8, whereas silencing ARMH4 enhances cytokine secretion. These observations suggest that ARMH4 plays a protective role in maintaining podocyte integrity by regulating inflammatory signalling pathways, and its downregulation may contribute to the inflammatory milieu characteristic of CG [91].​

#### Monogenic podocytopathies

Primary monogenic podocytopathies encompass a spectrum of inherited disorders in which pathogenic variants in genes critical for podocyte architecture or signalling precipitate nephrotic syndrome, often within the first year of life. These conditions follow Mendelian inheritance and are typically resistant to corticosteroid therapy, necessitating early genetic diagnosis to guide management and avoid unnecessary immunosuppression. Monogenic cases can be due to mutations in one of >50 genes including *NPHS1*, *NPHS2*, *WT1*, *INF2*, *TRPC6*, *ACTN4*, or *phospholipase C epsilon 1* (*PLCE1*) (Table 2) [67, 92, 93]. Collectively, mutations in *NPHS1*, *NPHS2*, *WT1*, *LAMB2*, and *PLCE1* account for approximately two‐thirds of congenital and infantile-onset nephrotic syndrome cases [94].

Nephrin, encoded by *NPHS1*, is a fundamental slit-diaphragm adhesion molecule. Biallelic early truncation *NPHS1* mutations cause the ‘Finnish-type’ congenital nephrotic syndrome, manifesting with massive proteinuria and oedema at birth. Histologically, these patients exhibit diffuse mesangial expansion and generalized foot‐process effacement. More than 30 pathogenic *NPHS1* variants have been identified worldwide, with two founder nonsense mutations accounting for most cases in Finland [95].

Another critical protein is *NPHS2*, which encodes podocin, an integral component of the slit diaphragm. *NPHS2* mutations are the most common monogenic form in childhood and are associated with autosomal recessive FSGS. Podocin interacts with nephrin and CD2AP to maintain the structural integrity of the slit diaphragm. When podocin is deficient or dysfunctional due to mutations, the interactions between these key proteins are disrupted, leading to foot-process effacement, proteinuria, and glomerulosclerosis [96–99]. The resulting damage to the filtration barrier compromises kidney function, contributing to disease progression.

TRPC6, a calcium channel expressed in podocytes, also plays a pivotal role in FSGS [100]. Mutations in TRPC6 result in autosomal dominant FSGS, where altered calcium influx triggers pathological signalling in podocytes, leading to cytoskeletal remodelling, podocyte apoptosis, and glomerular sclerosis [101]. The gain-of-function mutations in TRPC6 increase calcium entry, which exacerbates podocyte injury and promotes glomerulosclerosis [102, 103]. This highlights the importance of calcium homeostasis in podocyte health and glomerular function.

Many of these genetic defects disrupt slit-diaphragm signalling or cytoskeletal dynamics. For example, *ACTN4* is a protein that binds actin filaments in podocytes. Mutations in *ACTN4* lead to autosomal dominant FSGS by destabilizing the actin cytoskeleton, disrupting podocyte foot processes, and impairing the GFB. These mutations result in increased podocyte motility and altered actin bundling, which contribute to cellular dysfunction and glomerular injury [104, 105].

MYO1E, a non-muscle myosin, plays a critical role in the maintenance of podocyte function. Mutations in MYO1E disrupt the actin cytoskeleton and impair podocyte motility, leading to foot-process effacement and progressive glomerulosclerosis [106, 107]. This underscores the importance of myosin-mediated cytoskeletal dynamics in preserving podocyte integrity and glomerular filtration. The APOL1 gene, notably associated with FSGS in individuals of African descent, encodes apolipoprotein L1 [108]. Variations in APOL1 (G1 and G2 alleles) induce mitochondrial dysfunction in podocytes, triggering a pathological stress response that exacerbates podocyte injury and promotes FSGS [109, 110]. This highlights the role of genetic evolution in predisposing individuals to kidney disease through altered mitochondrial function.

Mutations in LMX1B, a transcription factor essential for podocyte function, are implicated in FSGS in patients with Nail–Patella syndrome [111]. LMX1B regulates critical genes involved in podocyte function and GBM integrity. Loss of LMX1B function leads to disruption of these pathways, resulting in podocyte injury and glomerulosclerosis [30]. CD2AP, a scaffolding protein that links nephrin to the actin cytoskeleton, is crucial for maintaining podocyte integrity. Mutations in CD2AP result in defective podocyte function, foot-process effacement, and the development of glomerulosclerosis, further implicating the actin cytoskeleton in FSGS pathogenesis [112].

The WT1 transcription factor is indispensable for podocyte differentiation and maintenance of slit-diaphragm proteins such as nephrin. Heterozygous WT1 mutations underlie Denys–Drash syndrome, which presents with diffuse mesangial sclerosis, steroid-resistant nephrotic syndrome, Wilms tumour risk, and urogenital anomalies. Murine models of Denys–Drash demonstrate that WT1 deficiency in podocytes leads to loss of differentiation markers, cytoskeletal reorganization, and glomerulosclerosis, recapitulating human disease pathology [113]. A related WT1 disorder, Frasier syndrome, results from donor splice-site mutations in intron 9 of the WT1 gene that disrupt the normal +KTS/−KTS isoform ratio, leading to later-onset FSGS and 46, XY gonadal dysgenesis with streak gonads and high risk of gonadoblastoma [114, 115].

Pierson syndrome, caused by biallelic LAMB2 mutations, exemplifies a GBM‐linked monogenic podocytopathy. Laminin β2 is a key component of the GBM essential for podocyte adhesion; its absence results in congenital nephrotic syndrome accompanied by ocular abnormalities such as microcoria and neurodevelopmental delay. Kidney biopsies reveal GBM thickening, mesangial expansion, and extensive foot-process effacement, mirroring diffuse mesangial sclerosis [116, 117].

More recently, recessive mutations in PLCE1, encoding phospholipase Cε1, have been recognized as causes of early‐onset nephrotic syndrome with diffuse mesangial sclerosis or FSGS. PLCE1 regulates signal transduction pathways crucial for podocyte survival and cytoskeletal dynamics; patients with PLCE1 mutations present in infancy with steroid-resistant proteinuria and rapid progression to kidney failure [93, 118].

Given that monogenic aetiologies underlie most congenital and infantile‐onset nephrotic syndromes and a large proportion of childhood onset SRNS, routine genetic screening is recommended for all children presenting with steroid‐resistant nephrotic syndrome before initiating prolonged immunosuppression. Early identification of an underlying monogenic cause informs prognosis, directs therapy (e.g. timely listing for transplantation), and facilitates genetic counselling for affected families [119] (Table 2).

### Secondary podocytopathies

Secondary podocytopathies arise from systemic disorders or exogenous insults that compromise podocyte integrity through indirect mechanisms, in contrast to primary podocytopathies that originate from intrinsic defects within podocytes themselves or direct podocyte insults [120]. Key etiological categories will be discussed next (Table 3).

Metabolic and hemodynamic stressors—such as diabetes mellitus, obesity, and systemic hypertension—increase glomerular capillary pressure and single‐nephron hyperfiltration, inducing podocyte hypertrophy and exposing cells to biomechanical stress [121, 122]. Chronic hyperglycaemia and dyslipidaemia further exacerbate podocyte injury through oxidative stress, endoplasmic reticulum (ER) stress, lipotoxicity, mTORC1‐mediated insulin resistance, and mitochondrial dysfunction, culminating in podocyte apoptosis and albuminuria [123–127]. In hypertensive nephrosclerosis, elevated glomerular capillary pressure imposes mechanical strain that activates TRPC6‐mediated calcium influx, leading to cytoskeletal collapse and foot‐process effacement [128, 129]. In lupus nephritis, a complication of systemic lupus erythematosus, features immune-complex deposition and anti‐dsDNA autoantibodies directly bind podocyte antigens, triggering complement activation and NF-κB signalling pathways, which destabilize the slit diaphragm and promote podocyte dysfunction [130–132]. All these maladaptive responses drive segmental glomerulosclerosis and effacement of podocyte foot processes: the histologic hallmarks of secondary FSGS [133, 134].

Autoimmune conditions such as systemic lupus erythematosus (SLE) and ANCA-associated vasculitis induce subendothelial and subepithelial immune-complex deposition along with complement activation, triggering local release of cytokines (e.g. TNF-α, IL-1β) that disrupt podocyte slit diaphragms and promote apoptosis [135, 136]. In membranous nephropathy, persistent subepithelial deposition of IgG-containing immune complexes thickens and spikes the GBM, activates the membrane attack complex (C5b-9), and induces reactive oxygen species in podocytes, leading to foot-process effacement [137, 138].

Non-Mendelian genetic variants can modulate individual risk. Notably, high-risk *APOL1* genotypes—prevalent in individuals of African descent—encode gain-of-function ion channels that become pathologically activated under inflammatory conditions [139, 140]. These alleles significantly increase susceptibility to secondary forms of FSGS or CG, particularly in the context of ‘second hits’ such as interferon exposure or viral infections [141].

Drug- and toxin-induced podocytopathies arise from the direct cytotoxic actions of various pharmacologic and recreational agents on podocytes, leading to proteinuria and secondary forms of FSGS or CG. Interferon-α therapy, used in viral hepatitis and certain malignancies, upregulates APOL1 expression in podocytes and induces apoptosis, often accompanied by characteristic tubuloreticular inclusions on electron microscopy [61, 142]. Intravenous bisphosphonates such as pamidronate are linked to collapsing FSGS, producing extensive foot-process effacement and podocyte detachment that may partially reverse following drug withdrawal [143–145]. Calcineurin inhibitors (cyclosporine A, tacrolimus) modulate synaptopodin phosphorylation and actin dynamics but, at higher concentrations or prolonged exposure, can provoke synaptopodin degradation by cathepsin L and consequent cytoskeletal disorganization and apoptotic signalling in cultured podocytes [146]. mTOR inhibitors (e.g. sirolimus, everolimus) disrupt autophagic flux and slit-diaphragm-associated protein expression (synaptopodin, podocin, nephrin), contributing to podocyte dysfunction and nephrotic-range proteinuria in transplant recipients [147].

Similarly, certain viral pathogens can directly injure podocytes, precipitating collapsing or sclerotic lesions. HIV-1 infects podocytes—despite limited CD4 expression—via alternative entry pathways, leading to expression of viral proteins (Nef, Vpr), dysregulation of host signalling, podocyte dedifferentiation, proliferation, and cytoskeletal collapse characteristic of HIV-associated nephropathy [148]. Parvovirus B19 DNA has been detected in renal biopsies from patients with collapsing FSGS, where viral infection correlates with diffuse foot-process effacement and podocyte hypertrophy, supporting a causative role in glomerular injury [149]. More recently, SARS-CoV-2 has been implicated in COVID-19–associated nephropathy; ACE2 expression on podocytes permits viral entry, while the associated cytokine storm (e.g. interferon, TNF-α) exacerbates podocyte apoptosis and foot-process effacement, culminating in collapsing lesions [150].

In systemic deposition disorders—such as AL amyloidosis [151], light-chain deposition disease (LCDD) [152], and cryoglobulinemia [153]—abnormal proteins aggregate within the GBM and mesangial matrix, distorting their architecture and mechanically disrupting podocyte adhesion. In AL amyloidosis, amyloid fibrils deposit predominantly in the GBM and mesangium, leading to isolated nephrotic-range proteinuria and, on biopsy, extensive GBM thickening with podocyte foot-process effacement [154]. Similarly, LCDD is characterized by nodular mesangial expansion and GBM thickening from monoclonal light-chain deposits, which precipitate podocyte dysfunction and overt nephrotic syndrome [155]. Cryoglobulinaemic glomerulonephritis features subendothelial and mesangial immune-complex aggregates that provoke complement activation, inflammatory injury, and secondary podocyte loss manifesting as proteinuria and mild nephritic features [153]. Podocyte detachment—an early event in sclerosis—has been shown to mark the transition from reversible injury to irreversible glomerular scarring in these diseases [156].

Conditions that reduce nephron endowment—such as congenital renal hypoplasia, reflux nephropathy, or unilateral nephrectomy—force the remaining nephrons into compensatory hyperfiltration. The resultant glomerular hypertension and increased single-nephron filtration pressure subject podocytes to excessive biomechanical stretch and shear forces. In rodent models of uninephrectomy, early post-surgical increases in single-nephron GFR correlate with podocyte hypertrophy followed by detachment from the GBM [157]. Biomechanical studies highlight that tensile stress and fluid-flow shear stress on podocytes during hyperfiltration disrupt cell–matrix adhesion, triggering foot-process effacement and eventual cell loss [122, 158]. In paediatric unilateral nephrectomy cohorts, pronounced early hyperfiltration predicts long-term podocyte depletion and glomerulosclerosis [159]. These maladaptive structural adaptations culminate in segmental scars characteristic of secondary FSGS [160], with congenital hypoplasia similarly predisposing to hyperfiltration-mediated podocyte injury in early life [161].

In all these cases, management focuses first on controlling the underlying disease (strict glycaemic control in diabetes, antiviral therapy for infection, immunosuppression in lupus, among other disease-specific approaches), and second on protecting podocytes. Therapies include renin–angiotensin blockade or SGLT2 inhibitors to reduce hyperfiltration, and immunomodulators when immune complexes are causal. Secondary podocytopathies do not respond to standard steroid or cytotoxic regimens aimed at primary podocyte disease unless the precipitant is addressed.

## CURRENT LANDSCAPE AND INNOVATIONS IN PODOCYTOPATHY GENE THERAPY

Our previous review has outlined a broad spectrum of viral and non-viral platforms for renal gene delivery—ranging from adeno-associated and adenoviral vectors to lipid nanoparticles and hydrodynamic methods—each with distinct advantages in tropism, payload capacity, and immunogenicity [8]. Building on this foundation, the present review zeroes in on gene-therapy approaches for podocytopathies, where AAV-based systems currently dominate thanks to innovations in capsid testing and transcriptional targeting that enhance podocyte specificity and transduction efficiency [162]. Emerging strategies—such as dual-vector approaches to overcome AAV’s payload limit, microRNA-based detargeting to minimize off-target expression, and rational promoter design—to usher these therapies from preclinical promise towards clinical reality are also discussed.

As mentioned, adeno-associated viruses (AAVs) have rapidly become the leading platform for podocyte-targeted gene therapy, thanks to their low immunogenicity, capacity for long-term expression in podocytes (given podocytes are terminally differentiated), and an expanding arsenal of capsid engineering and promoter-optimization strategies. AAV-mediated gene rescue in monogenic podocytopathies has been demonstrated by Wen Ding *et al.*, firmly establishing podocytes as tractable *in vivo* targets [163]. Here, in both inducible podocin–knockout and knock‐in mouse models that faithfully recapitulate the NPHS2 monogenic nephrotic syndromes, systemic delivery of AAV2/9 carrying wild‐type NPHS2 under a minimal nephrin promoter restored podocin mRNA and protein at the slit diaphragm, reduced albuminuria by >75% at days 14–42 post-induction, markedly improved histological features of glomerulosclerosis, and extended median survival from 66 to 192 days. In parallel, *in vitro* studies revealed that the synthetic capsid AAV-LK03—especially when paired with a podocyte-specific promoter—achieves efficient transduction of human podocytes and corrects the R138Q podocin trafficking defect, demonstrating functional rescue of cell adhesion [163]. Systemic delivery of AAV2/9 vectors with a podocyte-specific promoter led to off-target transduction in highly perfused organs, most notably the liver and spleen. In the conditional podocin knock-in mice, tail-vein injection of AAV2/9 carrying the minimal human NPHS1 promoter did achieve podocyte expression, but RNAScope and qPCR indicated substantial extra-renal uptake. Of note, Nakai’s group have found that AAV2/9 transduces podocytes more effectively in an albuminuric mouse model compared with wild-type mice potentially due to increased permeability of the GBM, which highlights the importance of testing gene-therapy vectors in the context of diseased models [164].

High levels of vector genomes in the liver can trigger innate and adaptive immune responses, manifesting as elevated transaminases, complement activation, and even acute liver failure in severe cases [165]. Clinical trials of high-dose AAV gene therapies report hepatotoxicity rates up to 50%, necessitating prolonged immunosuppression to manage inflammation and prevent transgene loss [166]. Moreover, off-target expression in splenic and pulmonary tissues contributes to systemic cytokine release and may exacerbate multi-organ toxicity [167]. This off-target tropism reflects that systemic delivery of AAV2/9 naturally favours hepatocytes, so even with a podocyte-specific promoter, high vector doses can overwhelm promoter stringency and drive transcription in the liver and spleen. Therefore, devising new ways to minimize hepatic and splenic transduction, reduce immune activation, and improve the safety profile of podocyte-directed gene therapies is significant for translation to the clinic.

Strategies to mitigate these effects include transcriptional targeting (e.g. tissue-specific promoters or microRNA target sequences), capsid engineering to alter tropism, and peptide display to enhance renal selectivity. Incorporating liver-specific microRNA-122 target sites into AAV vectors substantially reduces unintended hepatic expression by promoting transcript degradation in hepatocytes, while preserving expression in non-hepatic tissues [168]. Similarly, engineered synthetic promoters as short as 120 base pairs can achieve highly specific transgene expression in photoreceptor cells—demonstrating up to 1000- to 10 000-fold greater specificity compared with off-target cell types, while maintaining potency comparable to widely used promoters such as CAG (CMV enhancer fused to chicken beta actin promoter) [169]. Altering the viral capsid itself can shift tropism away from the liver.

Capsid engineering of AAVs has not thus far identified an improved capsid for the podocyte. However, screening of known capsids and directed evolution using diverse libraries, e.g. peptide libraries inserted into the most relevant AAV capsid have identified some very efficient AAV capsids for targeting the tubules. First, a barcoded screen of 47 AAV capsids has identified AAV-KP1 as a robust serotype for the proximal tubules in mice and non-human primates, particularly when delivered directly to the renal pelvis. This directed injection approach of AAV-KP1 led to dramatic reduction in liver expression in both mice and non-human primates, while the presence of neutralizing antibodies that suppressed liver transduction had no effect on proximal tubule transduction(170). This finding was in a small number of animals, but it would be very important to confirm this to inform planning of clinical trials and patient inclusion criteria. Cross-species capsid library cycling has yielded new AAV serotypes (AAV.k13 and AAV.k20) with enhanced tropism mainly for proximal tubular cells in mice, pigs, and non-human primates, demonstrating translational potential [162]. In addition, a newly evolved AAV2 variant (AAV2-GEC) achieves highly specific glomerular endothelial transduction after systemic delivery, preventing antibody-mediated kidney injury in mice, however, the main hurdle for translation here would be to determine whether transduction is equally efficacious in human glomerular endothelial cells [170]. The identification of these novel capsids has broadened the landscape for delivery of gene therapies particularly for tubular disease. It would be beneficial to use these capsid development approaches to improve AAV gene delivery to the podocyte, particularly across different species, which will simplify the translational pathway.

To overcome challenges in AAV transduction of podocytes, Purespring Therapeutics (https://purespringtx.com) has used a local method of administration to deliver two novel AAV gene therapies, PS-001 [171] and PS-002 [172], to the podocyte in Gottingen minipigs. Using this delivery method, both AAV gene therapies transduce most porcine podocytes, with low numbers of transcripts detected outside the kidney, demonstrating specificity of the gene therapy.

## ADDRESSING NON-MONOGENIC PODOCYTOPATHIES BY MOLECULAR TARGETING

Although gene therapy has been most successful for gene addition or augmentation approaches in autosomal recessive disease, there are pathways in non-monogenic podocytopathies that could potentially be targeted specifically by a gene-therapy approach.

As discussed before, podocytopathies involve diverse molecular pathways, necessitating tailored therapeutic strategies. For instance, in MCD, podocyte-secreted angiopoietin-like 4 binds to β1-integrin and downregulates nephrin at the slit diaphragm [173]: neutralizing ANGPTL4 or delivering nephrin via AAV vectors could restore the filtration barrier and reduce proteinuria. FSGS driven by TRPC6 gain-of-function mutations results in aberrant calcium influx and calcineurin activation [174]; here, siRNA-mediated TRPC6 regulation or CRISPR-based correction could normalize calcium homeostasis. In APOL1-associated nephropathies, risk variants (G1/G2) induce lysosomal membrane permeabilization and NLRP3 inflammasome activation [175], highlighting the potential of APOL1-specific siRNA or small molecule inhibitors (e.g. Inaxaplin). Finally, in diabetic podocytopathy, chronic hyperglycaemia activates Rho-associated kinase 1, which promotes Drp1 recruitment to mitochondrial membranes and drives excessive fission that fragments the network and impairs ATP synthesis [176, 177]. In this case, enhancing mitofusin-2 (Mfn2) expression represents a novel and potentially effective strategy for restoring mitochondrial balance—one that remains largely unexplored. Mfn2 plays a critical role in regulating mitochondria–ER contacts (MAMs), maintaining mitochondrial morphology, and inhibiting PERK-mediated apoptosis in podocytes under diabetic conditions [178]. Although AAV-mediated delivery of Mfn2 remains to be explored in podocyte models of diabetic kidney disease, these findings suggest that promoting mitochondrial fusion through gene-therapy approaches could preserve bioenergetic function, reduce oxidative stress, and ultimately protect against podocyte loss.

In addition to monogenic podocytopathies, IgA nephropathy (IgAN) has recently emerged as a landmark example of non-monogenic, podocyte-focused gene therapy, validating the concept that precise targeting within the podocyte can be broadly applied to glomerular diseases. Systemic inhibition of the alternative complement pathway has been a successful approach in the treatment of IgA nephropathy e.g. Iptacopan [179]. The AAV-based candidate PS-002 from Purespring Therapeutics delivers a complement-regulating transgene directly to podocytes. In murine IgAN models, PS-002 reduced proteinuria, mesangial C3 deposition, inflammation, and fibrosis, while in minipigs it achieved sustained, glomerulus-wide expression with no reported safety concerns [180]. There are currently many novel therapies that are licensed or demonstrating promise in clinical trials for IgA nephropathy, but the strengths of AAV gene therapy are that a single injection would result in long-term expression and podocyte specificity. This therapy can potentially be used in combination with other therapies to maximally reduce albuminuria and reduce the risk of progressing to end-stage renal failure. Notably, the European Medicines Agency recently granted PS-002 orphan drug designation, underscoring its translational promise [181]. These results substantiate a generalizable paradigm: identify and modulate key podocyte-centric pathogenic pathways—such as complement dysregulation—to develop targeted gene therapies across a spectrum of podocytopathies.

Translating this conceptual framework into human trials, however, necessitates addressing several critical hurdles associated with AAV manufacturing, dosing, and immunogenicity.

Manufacturing clinical-grade AAV vectors for first-in-human trials remains a major challenge, requiring robust upstream and downstream processes, stringent quality control, and detailed analytical characterization to meet regulatory standards. Ideally, vector production for Phase I/II studies should be carried out in the same facility intended for commercial-scale manufacturing to avoid complications associated with technology transfer and process comparability [182].

Accurate dose determination is critical to balance therapeutic efficacy, minimize off-target toxicity, and control cost of goods. Notably, high systemic doses of AAV have been associated with thrombotic microangiopathy in other clinical contexts (e.g. spinal muscular atrophy trials) [183, 184]. For renal gene therapy, a localized delivery approach could enable lower vector doses, reduced systemic exposure, and improved safety margins. To meet regulatory expectations (FDA/EMA), such delivery and toxicity profiles will probably need to be validated in large-animal models.

Pre-existing immunity to AAV capsids also poses a major hurdle, particularly for potential re-dosing, which is generally avoided. Therefore, ensuring durable and stable transgene expression is essential for long-term efficacy [185]. Encouragingly, in hepatic AAV trials for haemophilia, stable transgene expression has persisted for up to 15 years [186]. Given that podocytes have extremely low cellular turnover compared with hepatocytes, there is a strong rationale to expect that a single administration of podocyte-targeted AAV therapy could provide lifelong benefit.

## TECHNICAL CHALLENGES TO IMPROVE THE POTENTIAL OF GENE THERAPY

Although AAV is a highly established gene-therapy vector for *in vivo* gene therapy, one of its main limitations is its packaging capacity. This limits the use of gene editing machinery such as CRISPR–Cas9 approaches and large genes as this would require the delivery of more than one vector. Other possible vectors that could potentially package these larger genes include adenovirus vectors or nanoparticles, but both require further development to target kidney cells more efficiently.

For adenovirus, it was found that swapping the Ad19 fibre onto the adenovirus serotype 5 decreased liver transduction. Kidney-homing peptides (e.g. HTTHREP) were then inserted onto the Ad19p fibre and these modified Ad19p vectors showed undetectable β-galactosidase expression in the liver, spleen, heart, and lung, while achieving high-level transgene expression in glomerular and tubular compartments. Early biodistribution studies confirm that >95% of viral genomes avoid hepatic sequestration, highlighting the promise of peptide-directed capsid engineering for precision nephrology applications [187, 188]. One particularly effective approach is combining minimal liver-tropic capsids (e.g. AAVrh10 or AAVPHP.B) with *in vivo* phage-display-identified renal homing peptides, which selectively enhances podocyte uptake after systemic delivery [164].

Given the significant advances in gene delivery systems and genome editing technologies, there is immense untapped potential for developing targeted therapies to treat kidney diseases, some of which will be discussed next. With the right AAV-based delivery systems (different depending on the type of disease and mutation), various gene-therapy approaches including gene augmentation, CRISPR-based genome editing for targeted gene repair, and gene expression modulation can be employed, which has been comprehensively reviewed before [8]. Emerging CRISPR–Cas9 technologies now allow for precise correction of pathogenic variants. In WT1-associated Denys–Drash syndrome [189–191], for example, prime editing can rectify splice-site mutations in induced pluripotent stem cell-derived podocytes, restoring proper WT1 isoform expression. Base editing, by employing adenine or cytosine deaminase-fused Cas9, can potentially be used to correct NPHS2 R138Q in murine models without double-strand breaks, minimizing off-target effects [7, 190]. Antisense oligonucleotides (ASOs) targeting COL4A5 in Alport syndrome—a hereditary glomerular disease caused by mutations in type IV collagen genes leading to progressive kidney dysfunction, hearing loss, and ocular abnormalities—exemplify RNA therapy’s potential for treating podocytopathies [192]. As shown previously, ASOs can be used to induce exon skipping, thereby restoring the expression of functional COL4A5 protein. In preclinical studies, ASO treatment in mouse models of Alport syndrome resulted in improved renal function and delayed disease progression [193]. In the context of APOL1 nephropathy, AAVs have been used to selectively deliver siRNAs that silence G1/G2 alleles in primate models, reducing proteinuria [194]. mRNA therapy [195] also represents a promising strategy, with the potential to transiently restore nephrin expression in podocytes through delivery of codon-optimized NPHS1 transcripts: providing functional benefit while longer-term gene correction approaches are developed.

## CHALLENGES IN CLINICAL TRANSLATION

Efficiently delivering therapeutic payloads to podocytes is impeded by the kidney’s sophisticated filtration barrier and the polarized architecture of podocytes, which together restrict access of systemically administered vectors to the glomerular tuft. Ultrasound-mediated microbubble cavitation has been shown to transiently enlarge GBM pores and enhance uptake of nanoparticles and nucleic acids into renal cells in preclinical models, but further optimization is needed to ensure safety and reproducibility in larger animals and humans [196]. Even when vectors reach the kidney, neutralizing antibodies against common AAV serotypes can abrogate transduction, and adaptive immune responses against bacterial Cas proteins threaten the durability of CRISPR-mediated editing; strategies such as renal-pelvis injection or immunosuppressive regimens are under investigation to bypass pre-existing immunity and mitigate inflammatory clearance [164, 197]. Finally, podocytopathies are genetically and molecularly heterogeneous—ranging from monogenic *NPHS1* mutations in congenital nephrotic syndrome to complex polygenic risk in FSGS—necessitating patient stratification through comprehensive genomic, proteomic, and transcriptomic profiling to guide personalized vector design and target selection [198, 199]. The clearest biological targets for AAV gene replacement are the monogenic podocyte disorders; however, these represent rare or ultra-rare populations, making commercial investment and large-scale development less compelling. With the ability to target the podocyte directly, there is also a scope to introduce genes that can modulate glomerular biology in specific diseases. A compelling example is IgA nephropathy, where local expression of complement inhibitors could attenuate disease activity specifically within the glomerulus.

## FUTURE DIRECTIONS

Patient-derived kidney organoids now faithfully recapitulate key aspects of glomerular biology—including slit-diaphragm assembly and podocyte polarization—and can be generated at scale for high-throughput screening of vector tropism, dose–response, and off-target toxicity, accelerating lead candidate selection [200]. Integrating multi-omics datasets (genomics, proteomics, epigenomics, and spatial transcriptomics) from patient biopsies and organoids is uncovering novel pathogenic nodes—such as mechanosensitive ion channels (PIEZO1/2) in hypertensive podocytopathy—that represent actionable therapeutic targets beyond classical nephrin replacement [201].

Combinatorial regimens that pair allele-specific APOL1 silencing with established pharmacologics such as SGLT2 inhibitors hold potential for synergistically reducing glomerular hyperfiltration, oxidative stress, and proteinuria, offering both acute and long-term protection of podocyte integrity.

Preclinical evidence that AAV gene therapy to the podocyte can be used to successfully treat monogenic disease as well as non-monogenic disease has been demonstrated. This has progressed to large-animal model studies with identification of a successful delivery routes with high podocyte transduction efficiency and specificity. The benefits of AAV therapy over conventional small molecules is that one single injection will result in long-term expression in this terminally differentiated cell, and that off-target effects can be mitigated as the therapy has been designed to express solely in the podocyte. The next steps are to bring this into the frame of reality for patients.

Gene therapy offers transformative potential for podocytopathies by targeting their genetic basis, particularly with rapid gene sequencing enabling precise patient stratification. While preclinical successes underscore its viability, clinical translation requires overcoming delivery inefficiencies, immune challenges, and disease heterogeneity. A multidisciplinary approach, integrating genomics, bioengineering, and nephrology, will be essential to realize precision therapies for these complex disorders.

## Data Availability

No new data were generated or analysed in support of this research.
