# Correction to: Molecular mechanisms in podocytopathies: finding suitable targets for a new era of glomerular gene therapy

**DOI:** 10.1093/ckj/sfag314

**Published:** 2026-10-01

This is a correction to: Nadia Tavakolidakhrabadi, Wen Y Ding, Gavin I Welsh, Moin A Saleem, Molecular mechanisms in podocytopathies: finding suitable targets for a new era of glomerular gene therapy, *Clinical Kidney Journal*, Volume 19, Issue 1, January 2026, sfaf332, https://doi.org/10.1093/ckj/sfaf332

In the originally published version of this manuscript, the grant number was omitted from the ‘Funding’ section.

The corrected ‘Funding’ section reads:

“MAS and GIW have received funding from Kidney Research UK, Nephrotic Syndrome Trust and UKRI Medical Research Council (grant number: MR/R013942/1).

